# Thermo‐kinetic analysis space expansion for cyclophilin‐ligand interactions – identification of a new nonpeptide inhibitor using Biacore™ T200

**DOI:** 10.1002/2211-5463.12201

**Published:** 2017-02-23

**Authors:** Martin A. Wear, Matthew W. Nowicki, Elizabeth A. Blackburn, Iain W. McNae, Malcolm D. Walkinshaw

**Affiliations:** ^1^ The Edinburgh Protein Production Facility (EPPF)Wellcome Trust Centre for Cell Biology (WTCCB)University of EdinburghUK

**Keywords:** cyclophilin‐A, inhibitor, nonpeptide, surface plasmon resonance, thermodynamics

## Abstract

We have established a refined methodology for generating surface plasmon resonance sensor surfaces of recombinant his‐tagged human cyclophilin‐A. Our orientation‐specific stabilisation approach captures his‐tagged protein under ‘physiological conditions’ (150 mm NaCl, pH 7.5) and covalently stabilises it on Ni^2+^‐nitrilotriacetic acid surfaces, very briefly activated for primary amine‐coupling reactions, producing very stable and active surfaces (≥ 95% specific activity) of cyclophilin‐A. Variation in protein concentration with the same contact time allows straightforward generation of variable density surfaces, with essentially no loss of activity, making the protocol easily adaptable for studying numerous interactions; from very small fragments, ~ 100 Da, to large protein ligands. This new method results in an increased stability and activity of the immobilised protein and allowed us to expand the thermo‐kinetic analysis space, and to determine accurate and robust thermodynamic parameters for the cyclophilin‐A–cyclosporin‐A interaction. Furthermore, the increased sensitivity of the surface allowed identification of a new nonpeptide inhibitor of cyclophilin‐A, from a screen of a fragment library. This fragment, 2,3‐diaminopyridine, bound specifically with a mean affinity of 248 ± 60 μm. The X‐ray structure of this 109‐Da fragment bound in the active site of cyclophilin‐A was solved to a resolution of 1.25 Å (PDB: 5LUD), providing new insight into the molecular details for a potential new series of nonpeptide cyclophilin‐A inhibitors.

**Abbreviations**
CsA: cyclosporin‐A
DLS: dynamic light scattering
EDC: 1‐ethyl‐3‐(3‐diaminopropyl) carbodiimide hydrochloride
His‐CypA: His‐tagged cyclophilin‐A
IMAC: immobilised metal affinity chromatography
ITC: isothermal titration calorimetry
NHS: *N*‐hydroxysuccinimide
NTA: nitrilotriacetic acid
PDB: Protein Data Bank
RU: response units
SEC‐MALS: size‐exclusion chromatography multiangle light scattering
SPR: surface plasmon resonance

Over the course of the last decade surface plasmon resonance (SPR) systems, along with their control and analysis software, have become easier and simpler to operate, to such an extent that they are almost universally available in laboratories for the characterisation of biomolecular interactions and small molecule drug‐discovery/hit validation studies 1, 2, 3, 4, 5, 6, 7, 8, 9. The major advantage of SPR instrumentation is that it allows the measurement of kinetic and affinity parameters and derivation of thermodynamic data specifically associated with complex formation and dissociation. Such thermodynamic profiling 10, 11, 12 can greatly enhance the correlation of solution‐binding measurements with structural features. This, in turn, allows the assignment of proportional energetic contributions to individual functional groups involved in the formation of a complex; the whole basis of structure‐based approaches to engineered therapeutics and drug design 9, 13, 14, 15. The ready availability and ease of use of the technology has to be tempered with a vigorous approach to validation of the surface being studied, especially for nonexperts. It is comparatively straightforward to produce an ‘active’ sensor surface that displays a ‘binding response’. However, in many SPR studies the experimental design lacks appropriate optimisation, and the data generated often inappropriately interpreted and/or analysed 16, 17, 18, 19. This unfortunately leads to the derivation of affinity and kinetic parameters that are not physiologically appropriate to the protein/system being studied, and poor correlation with the literature data from alternative approaches.

We have developed a simple and refined methodology for generating highly stable and active SPR sensor surfaces of recombinant human his‐tagged cyclophilin‐A (His‐CypA). Surfaces created by this approach produce values for the kinetic, equilibrium and thermodynamic parameters for the binary cyclophilin‐A–cyclosporin‐A (CsA) complex that correlates extremely well with data determined by a multitude of other solution techniques 20, 21, 22, 23, 24, 25, 26, 27, 28, 29, 30. Cyclophilins are a large subfamily of isomerases that catalyse the conversion of prolyl‐cis/trans isomers in folding polypeptide chains [Peptidyl prolyl isomerase (PPIases), EC 5.2.1.8]. The most extensively studied member of the family is the cytoplasmic isoform CypA and its natural, low‐nanomolar affinity, immunosuppressive cyclic undecapeptide inhibitor CsA 31. As well as playing roles in protein folding, CypA appears to be a fundamental component in numerous, quite disparate, biological processes 32 including viral infections 33, 34, 35, 36, 37, 38, 39, 40, 41, responses to inflammation 42 and a growing number of proliferative cancers and malignancies 43, 44, 45. This has resulted in considerable interest in the development of nonimmunosuppressive, nonpeptide CypA inhibitors as mechanistic tools and potential drugs for various diseases.

Although the basic framework of the methodology described in this article is grounded on initial work developed in our lab, the new protocols have been very significantly, and rationally, optimised. Our orientation‐specific stabilisation approach captures his‐tagged proteins under ‘physiological conditions’ (150 mm NaCl, pH 7.5) on Ni^2+^‐charged nitrilotriacetic acid surfaces, which have been very briefly activated for primary amine‐coupling reactions. This produces very stable and active surfaces of His‐CypA. Simple alteration of protein concentration with the same contact time (30 s) allows variable density surfaces to be easily made, with no loss of specific activity. Surfaces can thus be easily tailored for the study of numerous interactions; ranging from very small fragments (~ 100 Da) through to large protein ligands. The streamlined procedure increased the stability and activity of the protein on the sensor surfaces and allowed us to greatly expand the thermo‐kinetic analysis space; from 5 °C to 44 °C. We subsequently generated much more detailed and robust sets of kinetic and thermodynamic parameters for the CypA–CsA interaction and identified and characterised a small fragment, 2,3‐diaminopyridine (Mw = 109 Da) that bound stoichiometrically to the active site of His‐CypA with a mean *K*_d_ of 248 ± 60 μm. The X‐ray structure of this fragment bound in the active site of CypA was also solved to a resolution of 1.25 Å [Protein Data Bank (PDB): 5LUD], providing new insight into the molecular details for potential development of a new series of nonpeptide CypA inhibitors.

## Materials and methods

### Materials

All chemicals used were of the highest grade available commercially.

### Plasmid construction, protein production and purification

The open reading frame encoding for full‐length human CypA (M1 – E165) was synthesised and codon optimised (GENEART) for expression in *Escherichia coli*, with a hexa‐his tag (underlined), linker and a TEV protease cleavage site (bold, underlined) fused to the N terminus (MSKYHHHHHHDYDIPTT**ENLYFQ/G**‐M1‐CypA). Standard GATEWAY^®^ methodology was used to generate an expression vector in pDEST™14 (ThermoFisher, Waltham, MA, USA). Recombinant protein was overexpressed and purified to homogeneity from OverExpress C41 BL21(DE3) *E. coli* (Lucigen, Middleton, WI, USA), grown shaking (260 r.p.m.) at 30 °C for 16 h in 50 mL of EnPresso media (BioSilta, St. Ives, Cambridgeshire, UK) containing carbenicillin (100 μg·mL^−1^). Cell pellets were resuspended in 20 mm NaH_2_PO_4_, pH 7.4; 500 mm NaCl; 20 mm imidazole; plus protease inhibitors at 10% w/v, and lysed at 6 °C by a single passage through a Constant Systems Cell Disruptor (1.1 kW TS Benchtop) set at 22 kpsi, followed by centrifugation at 50 000 ***g*** for 1 h at 4 °C. The supernatant was filtered (0.22 μm) and then subsequently loaded onto an ÄKTAXpress™ (GE Healthcare, Little Chalfont, Buckinghamshire, UK) system fitted with 5 mL HiTrap Ni^2+^‐IMAC FF (GE Healthcare) and HiPrep S200 26/60 HR (GE Healthcare) columns, with standard configuration and settings for a two‐step affinity‐gel‐filtration protocol. A single 10‐column volume step to 100% IMAC Elution buffer was used for elution from the IMAC column. Buffers used for the purification were; IMAC Loading buffer: 20 mm NaH_2_PO_4_, pH 7.4; 500 mm NaCl; 20 mm Imidazole; 100 μm PMSF. IMAC Elution buffer: 20 mm NaH_2_PO_4_, pH 7.4; 500 mm NaCl; 500 mm Imidazole. Gel‐Filtration Buffer: 10 mm NaH_2_PO_4_, pH 7.5; 150 mm NaCl; 50 μm EDTA. His‐CypA was concentrated to 100 μm and stored at 4 °C in Gel‐Filtration Buffer or processed for X‐ray crystallography (see below). The standard culture and purification conditions described above result in a final yield of 19.8 mg, per litre equivalent, of ≥ 95% pure (judged by SDS/PAGE) His‐CypA.

### Monodispersity and size analysis

Size‐exclusion chromatography (ÄKTAMicro™; GE Healthcare) coupled with UV, static light scattering and refractive index (RI) detection (Viscotec SEC‐MALS 20 and Viscotek RI Detector VE3580; Malvern Instruments, Malvern, Worcestershire, UK) were used to determine the absolute molecular mass of His‐CypA in solution. Multiple injections of 100 μL of 1 mg·mL^−1^ (47.5 μm) His‐CypA were run on a calibrated Superdex‐75 10/300 GL (GE Healthcare) size exclusion column pre‐equilibrated in 10 mm NaH_2_PO_4_, pH 7.5; 150 mm NaCl; 50 μm EDTA at 22 °C with a flow rate of 0.8 mL·min^−1^. Light scattering, RI and *A*_280 nm_ were analysed by a homo‐polymer model (omnisec software, v5.02; Malvern Instruments) using the following parameters for His‐CypA: ∂*A*_280 nm_/∂*c* = 0.71 AU·mL^−1^·mg^−1^, ∂*n*/∂*c* = 0.185 mL·g^−1^ and buffer RI value of 1.334. Mass distribution analysis of the pure His‐CypA protein solutions by dynamic light scattering (DLS) was performed on a Zetasizer APS (Malvern Instruments) with five repeat runs of 60 μL (1 mg·mL^−1^) in 10 mm NaH_2_PO_4_, pH 7.5; 150 mm NaCl; 50 μm EDTA, at 22 °C, with a 120‐s equilibration.

### Peptidyl prolyl isomerase assay

This assay determines the rate of the *cis* to *trans* conversion of the peptidyl‐prolyl amide bond in the substrate *N*‐succinyl‐Ala‐Ala‐Pro‐Phe‐*p*‐nitroanilide (AAPF‐pNA). Selective hydrolysis of AA*trans*PF‐pNA by α‐chymotrypsin releases *p*‐ntitroaniline, the accumulation of which is monitored by absorbance at 400 nm. AAPF‐pNA, in 470 mm LiCl; 2,2,2‐trifluroethanol at 200 mm, was diluted to 4 mm in the same buffer immediately before use. Reactions were conducted at 12 °C on a Jasco V550 spectrophotometer with temperature control, in 50 mm HEPES, pH 8.0; 100 mm NaCl; 0.5 mm DTT, in a total volume of 1 mL, essentially as described 26, 28 with minor modifications. The final concentration of His‐CypA and AAPF‐pNA were 12.3 nm and 100 μm respectively. The apparent equilibrium dissociation constant, *K*_iapp_, for the inhibitor was determined by a least squares fit of Eqn (1) to plots of the initial reaction rate (background thermal isomerisation rate subtracted), *V*_0_ (in μm·s^−1^) versus the concentration of CsA in nm.

(1) $$V_{0}=V_{i}/2\times \left[\text{hCypA}\right]\times \left\{\left(\left[\text{hCypA}\right]-\left[\text{CsA}\right]-K_{\mathrm{iapp}}\right)+\sqrt{(}{\left(\left[\text{hCypA}\right]-\left[\text{CsA}\right]-K_{\mathrm{iapp}}\right)}^{2}+\left(4\times \left[\text{hCypA}\right]\times K_{\mathrm{iapp}}\right)\right)\}.$$

where *V*_i_ is the reaction rate at zero inhibitor concentration, [CsA] is the concentration of added CsA, *K*_iapp_ is the apparent equilibrium dissociation constant of CsA and [hCypA] is the concentration of His‐CypA. Correction for competition with sAA*cis*PF‐pNA substrate was performed using Eqn (2).

(2) $$K_{d}=\frac{K_{\mathrm{iapp}}}{1+(\left[\text{Sub}\right]/K_{m})},$$

where [Sub] is the initial AA*cis*PF‐pNA concentration (mean concentration = 54.7 ± 2.9 μm, ±SE, *n* = 9) and *K*_m_ is the Michealis constant of the substrate. For His‐CypA, the mean (±SE, *n* = 9) *K*_m_, *k*_cat_ and *k*_cat_/*K*_m_ values are 703 ± 72 μm, 6134 ± 233 s^−1^ and 8.06 × 10^6^ m ^−1^·s^−1^ respectively.

### Surface plasmon resonance equipment and reagents

Surface plasmon resonance measurements were performed using a BIAcore T200 instrument (GE Healthcare). Ni^2+^‐nitrilotriacetic acid and CM5 sensor chips, 1‐ethyl‐3‐(3‐diaminopropyl) carbodiimide hydrochloride (EDC), *N*‐hydroxysuccinimide (NHS) and ethanolamine (H_2_N(CH_2_)_2_OH) were purchased from GE Healthcare.

### Optimised capture/stabilisation of His‐CypA

Pure His‐CypA was immobilised and covalently stabilised on an nitrilotriacetic acid sensor chip using a refined protocol to that initially described 25. Following Ni^2+^ priming (30 s injection of 500 μm NiCl_2_ at 5 μL·min^−1^), dextran surface carboxylate groups were minimally activated by an injection of 0.2 m EDC; 50 mm NHS at 5 μL·min^−1^ for between 30 and 420 s. His‐CypA (at concentrations between 10 and 400 nm), in 10 mm NaH_2_PO_4_, pH 7.5; 150 mm NaCl; 50 μm EDTA was captured via the hexa‐his tag and simultaneously covalently stabilised on the surface by injection for 30 s, at 30 μL·min^−1^. Immediately following the capture/stabilisation step a single 15‐s injection of 350 mm EDTA and 50 mm Imidazole in 10 mm NaH_2_PO_4_, pH 7.5; 150 mm NaCl; 0.05% surfactant P20, 1% ethanol, at 30 μL·min^−1^ was used to remove noncovalently bound protein, followed by a 180‐s injection of 1 m H_2_N(CH_2_)_2_OH, pH 8.5 at 5 μL·min^−1^. The surface was further conditioned with a 600‐s wash with 10 mm NaH_2_PO_4_, pH 7.5; 150 mm NaCl; 0.05% surfactant P20, 1% ethanol; 1 mm EDTA at 100 μL·min^−1^. Specific protein activity was assayed by passing saturating amounts of CsA (1.0 μm) in 10 mm NaH_2_PO_4_, pH 7.5, 150 mm NaCl, 50 μm EDTA; 0.05% surfactant P20; 1% ethanol over the surface and was invariably greater than 95% for all densities generated.

### Direct covalent immobilisation of His‐CypA

His‐CypA was immobilised on a CM5 sensor chip utilising standard amine‐coupling chemistry. The sensor chip surface was activated by an injection of 0.2 m EDC; 50 mm NHS at 5 μL·min^−1^ for 420 s. His‐CypA (theoretical pI = 6.54) at 100 μg·mL^−1^ in 10 mm acetate, pH 5.8, and injected over the activated surface for 30 s. The amount of His‐CypA immobilised on the activated surface was typically between 580 and 800 response units (RU). After the immobilisation of the protein, a 420‐s injection at 5 μL·min^−1^, of 1 m H_2_N(CH_2_)_2_OH, pH 8.5, was used to quench excess active succinimide ester groups. The surface was further conditioned with a 600‐s wash with 10 mm NaH_2_PO_4_, pH 7.5; 150 mm NaCl; 0.05% surfactant P20, 1% ethanol at 100 μL·min^−1.^

### SPR thermodynamic experiments

The SPR single cycle kinetic experiments with CsA were performed, in triplicate at temperatures from 5 °C to 44 °C, in 3 °C increments. A threefold concentration series of CsA ranging from 2.47 to 200 nm, in 10 mm NaH_2_PO_4_, pH 7.5, 150 mm NaCl, 1 mm EDTA; 0.05% surfactant P20; 1% ethanol, was injected over the sensor surface, at 100 μL·min^−1^ with 60 s contact and dissociation times. The sensor surface was regenerated between experiments by dissociating any formed complex in running buffer for 1800 s at 100 μL·min^−1^. The apparent on‐rate, off‐rate and equilibrium dissociation constants were calculated from the sensorgrams by global fitting of a 1 : 1 binding model, with mass transport considerations, using analysis software (v2.02) provided with the Biacore T200 instrument (GE Healthcare).

### Thermodynamic calculations

For equilibrium thermodynamics, the van't Hoff equation states:

(3) $$\ln K_{d}=\left(\Delta H^{\circ }/RT\right)-\left(\Delta S^{\circ }/R\right),$$

where *K*_d_ is the equilibrium dissociation constant, *R* is the universal gas constant, *T* is the absolute temperature (K), Δ*H*° is the standard enthalpy change and Δ*S*° is the standard entropy change. Plots of ln*K*_d_ versus 1/*T* should be a straight line, with a slope of Δ*H*°/*R* and an intercept on the ordinate of Δ*S*°/*R*. However, this simplified relationship will not hold if the heat capacities of the reactants differs from the heat capacity of the complex and the relationship between ln*K*_d_ *versus* 1/*T* becomes

(4) $$RT\ln K_{d}=\Delta H_{T^{\circ }}^{\circ }-T\Delta S_{T^{\circ }}^{\circ }+\Delta {\text{Cp}}^{\circ }\left(T-T_{0}\right)-T\Delta {\text{Cp}}^{\circ }\ln\left(T/T_{0}\right),$$

where ΔCp° is the heat capacity change under standard conditions, and *T*_0_ is the reference temperature (298 K) 46. Data were fitted using kaleidagraph v4.1.3 software (Synergy Software, Reading, PA, USA).

### Fragment library and compound screening

The Scottish Hit Discovery Facility fragment library (670 bioactive fragments) was obtained from the University of Dundee Drug Discovery unit. Compounds were initially screened at 1 mm on a surface of 2866 RU of His‐CypA (flowcell‐2) and ~ 10 000 RU of covalently immobilised (using standard amine chemistry) human serum albumin (HSA; flowcell‐4) in 10 mm NaH_2_PO_4_, pH 7.5; 150 mm NaCl; 50 μm EDTA; 0.05% surfactant P20; 1% DMSO, at 30 μL·min^−1^ with a contact time of 30 s and dissociation time of 120 s. Solvent correction, carry‐over assessment and a 25% DMSO wash between samples were performed as standard. Cyclophilin‐A‐specific hits were further analysed with a twofold concentration series from 0.0625 to 1 mm in 10 mm NaH_2_PO_4_, pH 7.5; 150 mm NaCl; 50 μm EDTA; 0.05% surfactant P20; 1% DMSO, at 30 μL·min^−1^ with a 30‐s contact and dissociation time.

### Crystallisation and X‐ray crystallography

The His‐tag was removed from CypA by His‐TEV protease (1 : 100 ratio, TEV : His‐CypA with a 2‐h incubation period at 30 °C) and the protein repurified as above to remove free His‐tag, His‐TEV protease and any remaining uncleaved His‐CypA. Protein was concentrated to 25 mg·mL^−1^ in Gel‐filtration Buffer (see above), minus EDTA, and crystals were grown by vapour diffusion using the hanging‐drop method. CypA protein solution was mixed 1 : 1 with 100 mm Tris, pH 8.0; 22% PEG 8000 and crystals were obtained after 1 day at 4 °C. CypA:2,3‐diaminopyridine complex was prepared by transferring a coverslip containing a drop of native CypA crystals over a well solution of 35% PEG 8000 and equilibrating at 4 °C for 24 h. Following this, a single CypA crystal was transferred into a 1 μL drop of 100 mm Tris, pH 8.0; 35% PEG 8000; 100 mm 2,3‐diaminopyridine, and allowed to soak for 60 s. The crystal (~ 0.2 mm in length) was then immediately mounted in a 0.2‐mm cryo‐loop (Hampton Research, Aliso Viejo, CA, USA) and flash cooled in liquid nitrogen, with soaking solution acting as cryoprotectant. All diffraction data were collected at 100 K at the Diamond Light Source (DLS), on station I02. Data processing was carried out using mosflm 47 and aimless 48. The isomorphous structure of cyclophilin‐A in complex with the dipeptide Gly‐Pro (PDB ID 4N1M) was used to solve the structure using the program refmac, 49 part of the ccp4 50 suite of programs. Refinement and model building were performed using the program coot 51.

### Data accessibility

Atomic coordinates have been deposited in the Brookhaven Protein Data Bank under the accession number PDB: 5LUD.

### Miscellaneous

The molecular weights of CsA, His‐CypA and 2,3‐diaminopyridine are 1202.12, 21 040 and 109 Da respectively. Protein concentration was determined by *A*_280_ measurement and the extinction coefficient 14 690 m ^−1^·cm^−1^.

## Results and Discussion

**Figure 1:**
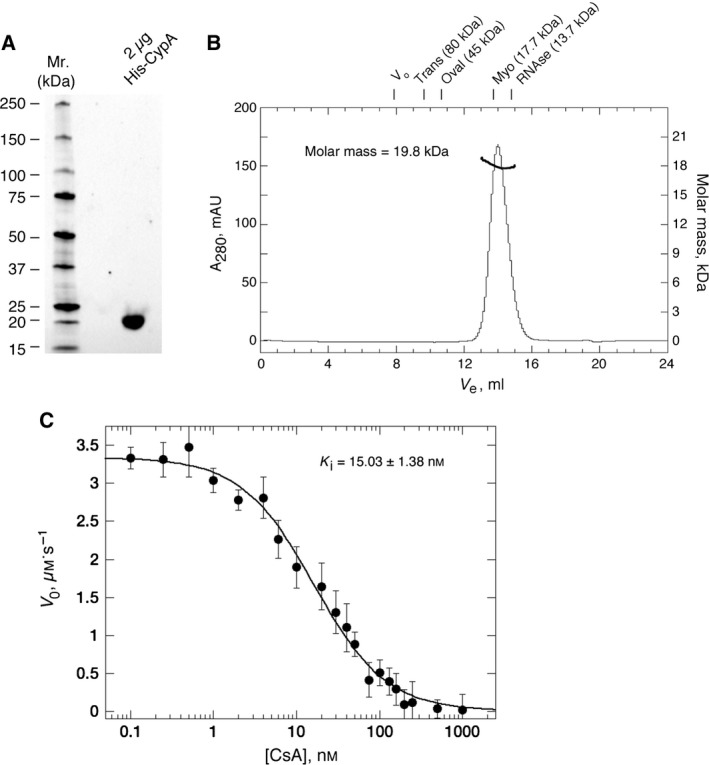
(A) Ultrapure, monodisperse and highly active protein is used for sensor‐surface generation. (A) 4–15% acrylamide SDS Stain‐free TGX gel (BioRad, Hercules, CA, USA) illustrating the final purity of His‐CypA (2 μg total protein). Standards are shown to the left. (B) Size‐exclusion chromatography (ÅKTA‐Micro; GE Healthcare) coupled with UV, static light scattering and RI detection (Viscotec SEC‐MALS 20 and Viscotek RI Detector:VE3580; Malvern Instruments), were used to determine the molar mass of His‐CypA in solution. About 100 μL of 1 mg·mL^−1^ His‐CypA was run on a Superdex‐75 10/300 GL (GE Healthcare) size exclusion column pre‐equilibrated in 10 mm NaH_2_ PO_4_, pH 7.5; 150 mm NaCl at 22 °C, at 0.8 mL·min^−1^. Light scattering, RI and *A*_280 nm_ were analysed by a homo‐polymer model (omnisec software, v 5.1; Malvern Instruments) using the following parameters: ∂*A*/∂*c* at 280 nm 0.71 AU·mL^−1^·mg^−1^ and ∂*n*/∂*c* of 0.185 mL·g^−1^. His‐CypA protein elutes a single sharp peak with a correlative *R*_s_ of 1.82 ± 0.1 nm (mean ± SEM,* n* = 5). Elution positions for standards are shown above the chromatograph. The molar mass average across the elution profile is 19.8 kDa with excellent monodispersity (Mw/Mn = 1.003). The theoretical molecular weight of His‐CypA is 21.04 kDa. DLS analysis (data not shown) also indicates high monodispersity for His‐CypA solutions with a mean *R*_h_ of 1.85 ± 0.17 nm (mean ± SEM,* n* = 5), a polydispersity index of ≤ 0.1, and a correlative molecular weight of between 19 and 21 kDa, consistent with a highly pure and monomeric protein solution. (C) Inhibition of His‐CypA's PPIase activity by CsA. Initial background corrected reaction (*V*_0_) rate in μm·s^−1^ is plotted versus the concentration of CsA in nm. Solid lines are a least squares fit of the data to Eqn (1) (see Material and methods). Each point is the mean ± SE,* n* = 3. The mean *K*_i_ value for CsA, at 12 °C, is 15.03 ± 1.38 nm.

One of the key criteria for successful SPR experiments is having access to very pure, monodisperse, active and stable protein. Our lab has previously generated protocols for the production and purification of numerous cyclophilins with very high purity and specific activity, both as tagged and untagged reagents, from bacterial sources 22, 26. We have streamlined a protocol for His‐CypA purification (see Material and methods) and Fig. 1 shows the typical high purity (≥ 95%), monodispersity and activity of the protein used in all our experiments. Size‐exclusion chromatography multiangle light scattering (SEC‐MALS) analysis was used to determine the molecular mass and monodispersity of purified His‐CypA in solution. On a Superdex‐75 10/300 GL (GE Healthcare) size‐exclusion column, His‐CypA protein elutes a single sharp and symmetrical peak with a correlative *R*_s_ of 1.82 ± 0.11 nm (Fig. 1B). The molar mass average across the elution profile is 19.8 ± 0.4 kDa with excellent monodispersity (Mw/Mn = 1.003). This is in excellent agreement with theoretical molecular weight of 21.04 kDa for monomeric His‐CypA. DLS (data not shown) analysis also indicates high monodispersity for His‐CypA solutions with a mean *R*_h_ of 1.85 ± 0.17 nm (mean ± SEM, *n* = 5), a polydispersity index of ≤ 0.1 and a correlative molecular weight of between 19 and 21 kDa, consistent with a highly pure and monomeric protein solution. This protein is also highly enzymatically active; Fig. 1C shows the typical inhibition of His‐CypA's PPIase activity by CsA. The mean *k*_cat_ value of 6134 ± 233 s^−1^ and the *K*_i_ value for CsA of 15.03 ± 1.38 nm (±SEM, *n* = 9) are again consistent with coherent protein with a very high specific activity 26, 28.

Covalent immobilisation of proteins via primary amine coupling is regularly the method of choice for generating SPR sensor surfaces with no baseline drift. It is a rapid and well‐established process and simple to implement. However, a significant proportion of proteins are incompatible with such covalent coupling strategies and as a consequence have aberrant or low binding activity, or are even completely inactive, upon immobilisation. This is certainly the case for the human cyclophilins, especially CypA, in our hands 26, 52. Alternative noncovalent capture methodologies like the His‐tag–Ni^2+^‐nitrilotriacetic acid interaction, are robust and allow for repeated immobilisation, stripping and regeneration of the surface 53. Nevertheless, these surfaces can often have low binding capacities, and/or exhibit a slow and continuous dissociation of the immobilised protein from the surface 26, 52, 53. Such a baseline drift, particularly prevalent with high protein immobilisation levels (due to loss of rebinding events) regularly required for small molecule work, can create problematic assessment of the binding kinetics. Previous work from our lab had investigated various orientation‐specific capture/stabilisation methods for the immunophilins, human CypA 25, 26 and human FK506‐binding protein (FKBP12) 52 that could not be actively immobilised by standard methods. Here, we report on the development of a simple and streamlined methodology for His‐CypA, that allows creation of very stable (in excess of 500 individual cycles without significant decay in saturating ligand concentration responses) and active SPR sensor surfaces (typically in excess of 95% specific activity). The approach captures and orients His‐tagged protein under ‘physiological’ ionic and pH conditions (minimising or eliminating any spurious electrostatic adsorption to the dextran matrix) on nitrilotriacetic acid surfaces previously charged with Ni^2^ and minimally activated for primary amine‐coupling reactions, prior to protein contact.

**Figure 2:**
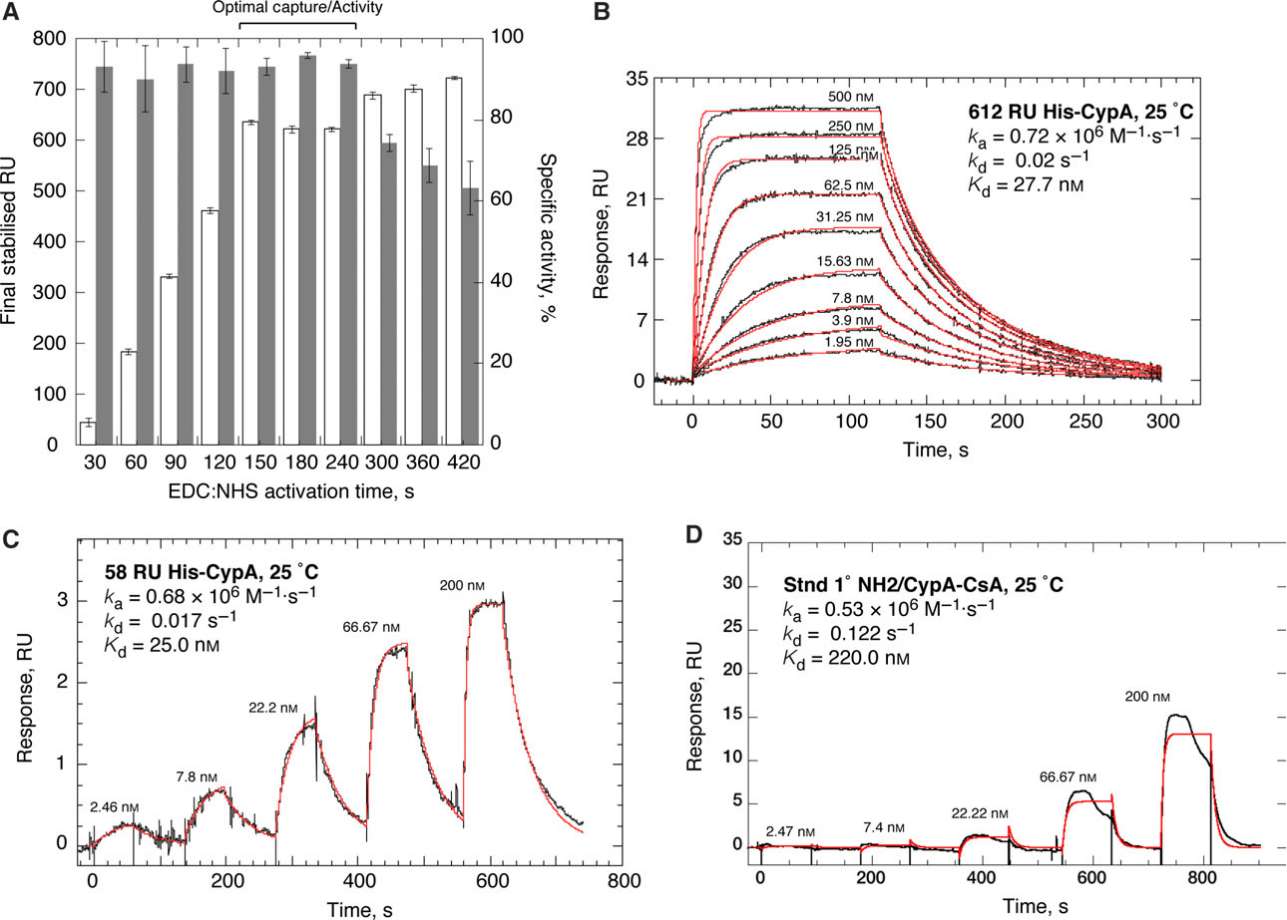
Optimisation of capture/stabilisation parameters on modified nitrilotriacetic acid‐sensor surfaces. (A) Graphical representation comparing the EDC–NHS activation time with the final levels of immobilised His‐CypA (white bars, left axis) and the corresponding specific activity of the surfaces (dark grey bars, right axis). Contact time in each experiment was 30 s with a 100 nm solution of His‐CypA. In all cases the experimental RU_max_ value was generated by passing 1.2 μm CsA in 10 mm NaH_2_ PO_4_, pH 7.5, 150 mm NaCl, 50 μm EDTA; 0.05% surfactant P20; 1% ethanol over the surface. (B) Representative reference corrected SPR sensorgrams (black), monitored on a surface with 612 RU of covalently stabilised His‐CypA (100 nm, 30 s of contact following 180 s of activation). A twofold dilution series of CsA, from 500 nm to 1.95 nm, was run at 25 °C in 10 mm NaH_2_ PO_4_, pH 7.5, 150 mm NaCl, 1 mm EDTA; 0.05% surfactant P20; 1% ethanol at 100 μL·min^−1^. The on‐ and off‐rate constants were by globally fitting (red) a 1 : 1 kinetic binding model to the sensorgrams using the analysis software (v2.02; GE Healthcare) supplied with the instrument. Mean values (*n* = 5, ±SEM) determined for the on‐rate (*k*_a_), off‐rate (*k*_d_) and equilibrium dissociation constants (*K*_d_) are (0.79 ± 0.06) × 10^6^ m ^−1^?s^−1^, 0.018 ± 0.004 s^−1^ and 22.8 ± 3.6 nm respectively. (C) Reference corrected SPR single‐cycle kinetic sensorgrams (black), monitored on a surface with 58 RU of covalently stabilised His‐CypA (10 nm, 30 s of contact following 180 s of activation). A threefold dilution series of CsA, from 200 to 2.46 nm, was run at 25 °C in 10 mm NaH_2_ PO_4_, pH 7.5, 150 mm NaCl, 1 mm EDTA; 0.05% surfactant P20; 1% ethanol at 100 μL·min^−1^. The on‐ and off‐rate constants were by globally fitting (red) a 1 : 1 kinetic binding model to the sensorgrams using the analysis software (v2.02; GE Healthcare) supplied with the instrument. Mean values (*n* = 3, ±SEM) determined for the on‐rate (*k*_a_), off‐rate (*k*_d_) and equilibrium dissociation constants (*K*_d_) are (0.73 ± 0.07) × 10^6^ m ^−1^?s^−1^, 0.018 ± 0.006 s^−1^ and 24.7 ± 5.3 nm respectively. (D) Reference corrected SPR single‐cycle kinetic sensorgrams (black), monitored on a surface with 780 RU of His‐CypA immobilised utilising standard amine coupling chemistry (100 μg·mL^−1^ His‐CypA in 10 mm acetate, pH 5.8, with a 30‐s contact time with the activated surface). A threefold dilution series of CsA, from 200 to 2.46 nm, was run at 25 °C in 10 mm NaH_2_ PO_4_, pH 7.5, 150 mm NaCl, 1 mm EDTA; 0.05% surfactant P20; 1% ethanol at 100 μL·min^−1^. The on‐ and off‐rate constants were by globally fitting (red) a 1 : 1 kinetic binding model to the sensorgrams using the analysis software (v2.02; GE Healthcare) supplied with the instrument. Mean values (*n* = 3, ±SEM) determined for the on‐rate (*k*_a_), off‐rate (*k*_d_) and equilibrium dissociation constants (*K*_d_) are (0.48 ± 0.1) × 10^6^ m ^−1^?s^−1^, 0.14 ± 0.1 s^−1^ and 291.7 ± 104 nm respectively.

We first determined the length of time the sensor surfaces were activated prior to his‐tagged protein capture was important for not only the final level of protein immobilisation but also critically the specific activity of the captured/stabilised molecules. The optimal time for EDC:NHS activation of the precharged Ni^2+^‐nitrilotriacetic acid sensor surface was invariably between 150 and 240 s, regardless of the concentration of protein used (Fig. 2A). Less than 150 s of activation time resulted in low and more variable levels of stabilised protein on the surface (Fig. 2A). Activating for longer than 240 s resulted in the specific activity dropping markedly below 80% (Fig. 2A) and the resultant binding parameters measured using such surfaces for the CypA–CsA interaction deviate markedly from accepted values for these constants. In our hands, neither the kinetics of binding, nor the specific activity of the surface, were affected to any significant extent by the amount of protein immobilised on the surface. Simply changing the concentration of His‐CypA from 10 to 400 nm, while keeping the divalent charging (30 s), activation (180 s), protein contact (30 s for all densities) and quenching (180 s) to minimal constant times, allowed us to both standardise the method and generate sensor surfaces with between ~ 50 and ~ 4000 RU of stabilised protein. Typical sensorgrams, monitored on intermediate (~ 600 RU) and extremely low (~ 50 RU) density surfaces of covalently stabilised His‐CypA binding to CsA, are shown in Fig. 2B,C. The on‐rate (*k*_+_), off‐rate (*k*_−_) and equilibrium dissociation constants (*K*_d_) are essentially identical for both surfaces. Mean values for *k*_a_, *k*_d_ and *K*_d_ at 25 °C, on intermediate density surfaces are (0.79 ± 0.06) × 10^6^ m ^−1^?s^−1^, 0.018 ± 0.004 s^−1^ and 22.8 ± 3.6 nm (Fig. 2B), respectively, compared to (0.73 ± 0.07) × 10^6^ m ^−1^?s^−1^, 0.018 ± 0.006 s^−1^ and 24.7 ± 5.3 nm (Fig. 2C), on low‐density surfaces. Our data here are in excellent agreement with the values for these biophysical parameters determined in solution studies by isothermal titration calorimetry (ITC) 20, 24, 25, fluorescence titration 23, 25, 26, 27, 29, SPR 25, 26, enzymatic analysis 23, 26, 28, 29 and NMR 21.

Capture via the His‐tag, under ‘physiological’ pH and salt concentration, reduces or eliminates any electrostatic adsorption to carboxmethyldextran matrix, while orienting the protein away from the sensor surface, and prevents any uncontrolled and spurious coupling reactions that are liable compromise the coherency of the immobilised protein. The covalent reaction on these surfaces is therefore focused and brief, and only occurs proximally to the His‐tag; likely either the N terminus itself or the lysine residue N‐terminal to the hexa‐his sequence in the construct. Similar rationales have been used for making sensor surfaces with whole cellular receptors 54 and His‐RGS proteins 55. In our hands, standard covalent coupling methods have never yielded satisfactory results for the cyclophilins. As previously discussed 26, the X‐ray structure of CypA 56 shows the majority of surface exposed lysine residues are located on the CsA‐binding face of the molecule giving it a predominantly basic character. Under the acidic solution conditions (even mildly acidic) required for the standard primary amine immobilisation process, the surface charge potential of CypA seems likely to force the molecule to orient with the CsA‐binding surface face‐down on the matrix, resulting in severe steric occlusion of this site for ligand access. A further consideration that contributes to low protein activity when using direct coupling approaches is an acidic pH shift‐dependent affinity loss that occurs quite rapidly with CypA. This behaviour has been observed in ITC, intrinsic fluorescence experiments with CypA and its ligands 24, 26, 30. The reasons and mechanism for this are not entirely clear, but it involves a combination of protein protonation and a resultant partial irreversible denaturation of the protein in solutions with a pH below ~ 5.5 that quickly leads to irreversible structural changes and aberrant binding behaviour 24, 26, 30.

The most effective conditions we have found in our laboratory so far for direct coupling of CypA are 100–120 μg·mL^−1^ protein in 10 mm acetate buffer, pH 5.8 (the predicted pI of the protein is 6.54), with a 30 s contact time on an activated CM5 sensor surface. Figure 2D illustrates the type of data typically generated from such surfaces. The protein immobilised on these sensors is very clearly compromised in terms of its biophysical state. Longer contact times reduce the already poor specific activity and anomalous ligand‐binding behaviour further. The specific activity is only around 35% and it decays quite rapidly. Furthermore, the interaction is clearly not 1 : 1 and has ill‐defined multiphasic kinetics (Fig. 2D). The mean values (mean ± SEM, *n* = 3) for *k*_a_, *k*_d_ and *K*_d_, at 25 °C, determined from fitting a 1 : 1 model, for which the data are clearly not well described, are (0.48 ± 0.1) × 10^6^ m ^−1^?s^−1^, 0.14 ± 0.1 s^−1^ and 291.7 ± 104 nm respectively. The affinity is 12–14‐fold weaker with significantly altered kinetics; *k*_d_ is 8–10‐fold faster and the *k*_a_ twofold slower, compared to the interaction assessed on surfaces of oriented and stabilised His‐CypA (compare Fig. 2B and D). This very clearly indicates that this is a protein surface that is far from being biophysically coherent and does not agree with the data from other solution studies. The accepted *K*_d_ range for the CypA:CsA interaction sits between 10 and 30 nm, with the variation arising from slight differences in the buffer systems and their ionic strength, the pH, the temperature of analysis and the specific analysis technique used in the particular study in question 20, 21, 23, 24, 25, 26, 27, 28, 29, 30. Surfaces of CypA generated by direct covalent immobilisation should really not then be utilised in any further experiments as they lack any rigorous correlative validation from any orthogonal techniques. Regrettably for the cyclophilins, and especially CypA, there are repeated examples in the literature where SPR sensor surfaces, all generated by standard primary amine coupling chemistry and showing very inappropriate and non‐native affinities and kinetics, have been used to screen/assay ligands. There are instances where such surfaces have been used without any assessment of appropriate activity with a positive control, others where surfaces are used despite the affinity for CsA being reported as being orders of magnitude weaker (10–50‐fold) than the normal expected range, and others still where the apparent binding affinities of ligands assessed by SPR do not correlate at all with any other assays employed in the study 57, 58, 59, 60. This is poor practice and at best, muddies the water for the field.

**Figure 3:**
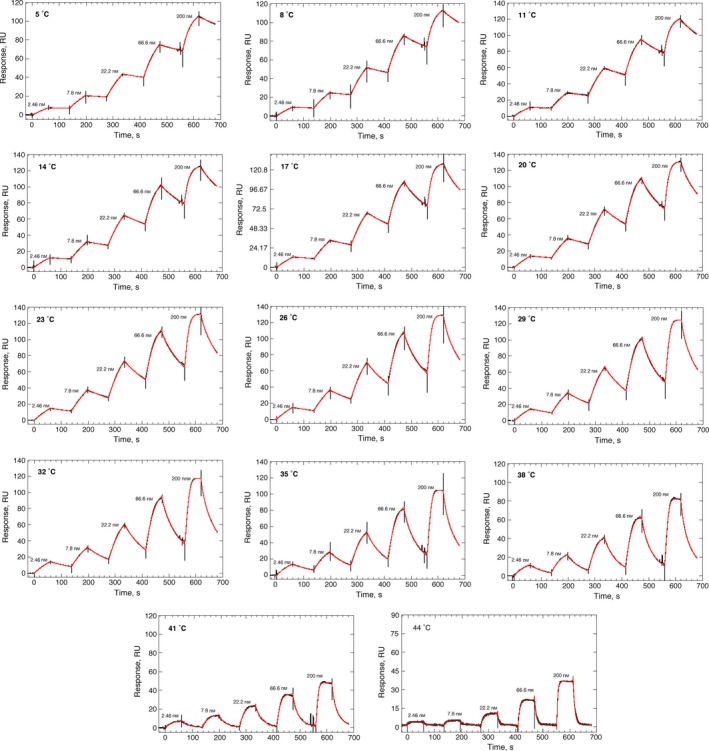
Effect of temperature on the binding of CsA to His‐CypA. Reference corrected SPR single‐cycle kinetic sensorgrams (black), monitored on a surface with 2378 RU of covalently stabilised His‐CypA (250 nm, 30‐s contact following 180‐s activation) for the indicated CsA concentrations (200–2.46 nm) from 5 °C to 44 °C in 3 °C increments. The on‐ and off‐rate constants were by globally fitting (red) a 1 : 1 kinetic binding model to the sensorgrams using the analysis software (v2.02; GE Healthcare) supplied with the instrument. Mean values (*n* = 3, ±SEM) determined for the on‐rate (*k*_a_), off‐rate (*k*_d_) and equilibrium dissociation constants (*K*_d_) are shown in Table 1.

We next assessed the ability of our His‐CypA surfaces to produce equilibrium thermodynamic data. Our streamlined surface generation clearly increased the coherency and stability of the protein on the sensor surfaces, allowing for a significant expansion of the thermo‐kinetic analysis space from 5 °C to 44 °C, compared to the previous range of ~ 16 °C to ~ 35 °C 25, 26. The protein on these new surfaces was stable at temperatures up to 44 °C and could be repeatedly raised to this temperature without significant loss of binding activity or deviation in the kinetics. Single‐cycle kinetic experiments with CsA were performed at temperatures ranging from 5 °C to 44 °C, in 3 °C increments, with a threefold dilution series of CsA from 2.45 to 200 nm. Figure 3 shows typical fitted data from a high‐density sensor surface and the *k*_a_, *k*_d_ and *K*_d_ values derived from this analysis shown in Table 1. This increased sensor surface stability permits analysis of ligand interactions at physiologically relevant temperatures, critical for proper exploration of the temperature effects on the affinity and kinetics in small molecule drug‐discovery studies. Additionally, the increased sensitivity and structural integrity of the protein surface also allowed us to routinely use lower concentrations of CsA, eliminating the solubility issues with CsA (a very hydrophobic molecule), and allowing extension by over 10 °C of the lower temperature analysis range.

**Table 1:** Temperature dependence of the kinetic and equilibrium affinity constants for CsA binding to recombinant human His‐CypA, determined by Biacore™ T200

Temp (°C)	*K*_d_ (nm)	*k*_a_ (m ^−1^·s^−1^)	*k*_d_ (×10^−3^ s^−1^)
5	5.5 ± 1.2	(0.22 ± 0.05)×10^6^	1.2 ± 0.08
8	7.1 ± 1.2	(0.27 ± 0.02)×10^6^	1.9 ± 0.2
11	7.6 ± 1.7	(0.33 ± 0.08)×10^6^	2.5 ± 0.1
14	9.6 ± 1.2	(0.42 ± 0.04)×10^6^	4.1 ± 0.2
17	12.2 ± 2.1	(0.47 ± 0.08)×10^6^	5.7 ± 0.2
20	14.3 ± 1.4	(0.59 ± 0.04)×10^6^	8.4 ± 0.4
23	20.4 ± 2.7	(0.61 ± 0.1)×10^6^	12.4 ± 0.6
26	27.6 ± 4.9	(0.67 ± 0.1)×10^6^	18.5 ± 1
29	38.4 ± 8.2	(0.77 ± 0.14)×10^6^	29.5 ± 2.1
32	52 ± 6.1	(0.83 ± 0.09)×10^6^	43.0 ± 1.0
35	75.1 ± 13	(1.1 ± 0.21)×10^6^	83.6 ± 3.4
38	90.1 ± 9.6	(1.3 ± 0.36)×10^6^	118 ± 3.6
41	117 ± 16.6	(1.8 ± 0.41)×10^6^	211 ± 7.8

Temp (°C)	van't Hoff analysis			
	Δ*G*° (kcal·mol^−1^)	Δ*H*° (kcal·mol^−1^)	*T*Δ*S*° (kcal·mol^−1^)	ΔCp (kcal·mol^−1^·K^−1^)
25 °C	−10.38 ± 0.21	−15. 50 ± 0.8	−5.12 ± 0.5	−0.41 ± 0.16

K_d_ values (mean ± SE, *n* = 3) were calculated using the formula K_d_ = *k*_d_/*k*_a_. van't Hoff parameters calculated from a fit of Eqn (4) to a plot of ln*K*_d_ versus 1/*T* in K (Fig. 3).

**Figure 4:**
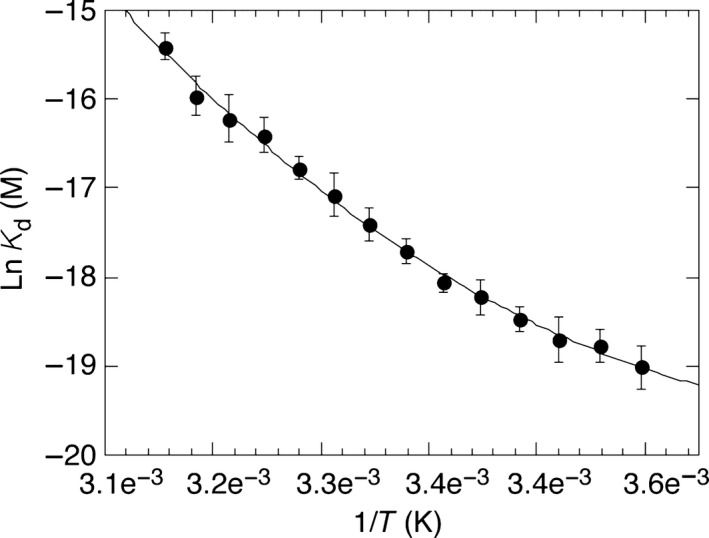
Thermodynamic characterisation of the CypA–CsA interaction by SPR Biacore™ T200. Plot of lnK_d_ *versus* 1/*T* in K. Data were fit (solid line) to Eqn (4) using kaleidagraph v4.1.3 software (Synergy Software). Thermodynamic parameters calculated from these data are shown in Table 1.

From 5 °C to 44 °C, the off‐rate for CsA is increased ~ 175‐fold compared to a ~ 9‐fold increase in the on‐rate (Table 1), resulting in a 23‐fold loss of affinity from ~ 5 to ~ 120 nm (Fig. 4A, Table 1). The van't Hoff plot of these data are very clearly nonlinear (Fig. 4) indicating a change in the heat capacity (ΔCp) of the system upon binding. A fit of Eqn (4) to these data (solid line, Fig. 4) gives the following thermodynamic parameters at standard temperature (25 °C); Δ*G*° = −10.38 ± 0.21 kcal·mol^−1^, Δ*H*° = −15.50 ± 0.8 kcal·mol^−1^, *T*Δ*S*° = −5.12 ± 0.5 kcal·mol^−1^; ΔCp = −0.41 ± 0.16 kcal·mol^−1^·K^−1^ (Table 1). Negative ΔCp values can be correlated with reduced solvent accessibility for nonpolar surfaces during complex formation. They are often also indicative of conformational complexity in one or other of the components of the reaction, with changes in conformation between free and complexed states being markedly different. Another important contributor to negative ΔCp values is the trapping of water molecules at the binding interface of the complex, often forming or contributing to a critical feature of the binding interaction surface, with each water having been calculated to contribute about – 0.05 kcal·mol^−1^·K^−1^ to the overall ΔCp 24, 61). The heavily enthalpic CypA–CsA interaction is not only primarily driven by hydrophobic and van der Waals interactions, but also critically, contacts that involve five well‐defined water molecules trapped at the binary complex interface 24, 61. This coupled to the significant entropic cost of complexing a large and flexible ligand such as CsA (a cyclic undecapeptide 62), explains the energetic parameters and the very obvious negative ΔCp in relation to the CypA–CsA complex structure 24, 25, 61, 62, 63, 64.

The thermodynamic parameters determined here agree exceptionally well with those measured in Fanghänel and Fischer's 24 detailed study of the CypA–CsA interaction by ITC, performed at 25 °C in 25 mm phosphate buffer at pH 7.5; Δ*G*° = −10.69 ± 0.11 kcal·mol^−1^, Δ*H*° = −14.7 ± 0.03 kcal·mol^−1^, *T*Δ*S*° = −3.8 kcal·mol^−1^; ΔCp = −0.44 ± 0.007 kcal·mol^−1^·K^−1^. The very small disparities arise due to subtle differences in the analysis platforms and varying contributions from solvation/desolvation effects, buffer ionisation/proton exchange effects (although essentially eliminated at pH 7.5) and conformational fluctuations (free diffusion *versus* surface immobilised) in the reactants during complex formation. There is now excellent convergence in the ΔCp values determined from this SPR study, those determined from rigorous ITC analysis 24 and those calculated from structure‐based calculations on the CypA:CsA binary complex; all are essentially ~ 0.4 kcal·mol^−1^·K^−1^ 24, 25, 61, 62, 63, 64. The agreement with solution, and label‐free studies, further supports the view that our His‐CypA SPR sensor surfaces, and the interaction of ligands with the protein, are unaffected by the immobilisation process and behave essentially as they would if free in solution and are representative of the correct physiological interaction. About 1000 RU of CypA on the surface corresponds to ~ 3 × 10^10^ protein molecules immobilised within a volume of ~ 1 × 10^14^ nm^3^. This gives an average intermolecular spacing between each His‐CypA molecule of at least 120 Å. 2000 RU gives spacing of ~ 85 Å and 3000 RU ~ 50 Å. None of these densities would have surface ‘crowding’ issues, and little possibility of heterogeneity arising due to steric occlusion by neighbouring molecules. This sparse and stable binding arrangement goes partway to explaining the excellent agreement in the values for the kinetic and equilibrium constants from our SPR data, the other solution techniques.

The BIAcore T200 instrument used in this study is by far the best platform for analysing such a system, both in terms of the signal‐to‐noise limits [baseline noise limits are < 0.03 RU (RMS), < 0.1 (RMS) and < 0.33 RU (RMS) for the T200, T100 and 3000 systems respectively; sourced directly from GE Healthcare product technical specification data] and the temperature control for analysis. A note here, the new BIAcore S200 system has better specifications for baseline noise, < 0.0015 RU (RMS), but with the same sensitivity for ligand detection as the T200, but we neither have access to nor have we tested the system.

The T200 system has an operating temperature range of 4–45 °C, and will actually achieve the limits of this range. The T100 has the same stated range limits, but this is heavily dependent on the ambient temperature the instrument is operated in. We know from experience that the system was able to only practically cool to ~ 18 °C below ambient and under standard laboratory conditions (even though the stated maximum is 20 °C). The 3000 instrument possesses a manufacturer's quoted range of 4–40 °C (less than the other two systems at the top end by 5 °C). Again, this is even more dependent on the ambient operating temperature the instrument the system is operated in. Our experience is that this system is able to only practically cool to ~ 15–16 °C below ambient (even though the stated maximum is 20 °C, this can only really be achieved reliably in environments with ambient temperatures of 18–19 °C). The superior functionality and sensitivity of the BIAcore T200 and the significantly increased activity and stability of the protein surfaces were primary factors in our ability to extend the thermo‐kinetic analysis space reliably.

The thermodynamic parameters generated across this expanded range by this new method are much more comprehensive and robust, and there is now a very good convergence in the energetic parameters for binding CsA, particularly for the ΔCp parameter, between the results reported here and those determined by ITC and structure‐based calculations 24, 62, where buffer systems have been matched. Notably, ΔCp is notoriously difficult to determine by noncalorimetric methods and a critical factor in formulating a complete understanding and rationalisation of the binding interface and structural features driving molecular recognition/complex formation; vital for rational ligand/drug design. This is especially so when using atomic resolution structures to guide the development of new ligands.

It is also noteworthy that the buffer system used here is phosphate‐buffered saline compared to HEPES‐buffered saline used previously. The thermodynamic parameters that have been determined by our lab and others change dependent on the buffer system used in the analysis. The changes can be small and sometime subtle, but genuine, and represent differences in the binding energetics, the interface contacts and the interactions between the protein and the solvent and solutes in it. Given the subtlety that clearly exists in the mechanism of binding for ligands in the active site of the cyclophilins and that this binding is ‘finessed’ by the solution conditions, it is important to have characterisation of the binding in multiple solvents/solution conditions. This allows for a more comprehensive understanding of the molecular contacts and how solution changes are likely to alter the interaction between the protein and the ligand. For example, the relatively small energetic changes that arise from trapping water molecules at the binding interface (~ −0.05 kcal·mol^−1^·K^−1^ per trapped water) and/or the conformational subtlety in the contacts with this pseudo binding interface and ligands could potentially add up to 10 s of nm difference in affinity, contributed from ΔCp alone. In addition to these differences in the binding energetics/molecular contacts, understanding these solution differences potentially allows for specificity to be built into future ligand development by exploiting the differences in the way isoforms and the protein–ligand complexes interact with the solvent. To do this efficiently and rationally, we feel it is crucial to characterise the interaction as fully as possible, in multiple solution environments that maintain physiological relevance.

The cyclophilins are validated drug targets for a number of diseases including HIV and HBV infection, malaria, recovery from ischemia, parasitic worm infection, immunosuppression and numerous proliferative cancers 23, 31, 32, 33, 34, 35, 36, 37, 38, 39, 40, 41, 42, 43, 44, 45. Our laboratory is particularly interested in finding new compounds and analogues that could provide new chemical scaffolds for the synthesis of families of peptidomimetic molecules with potential isoform‐specific inhibitory activity against these viral and parasite infections. Screening fragment (molecular weights in the 100–250 Da range) libraries, either by high‐throughput X‐ray crystallography, NMR or SPR has been established as a rationale that allows rapid and effective interrogation of key binding interactions on the protein, reproducibly and specifically 65, 66, 67. Even though a fragment's intrinsic potency is often very weak (*K*_d_ or IC_50_ values are typically in the high 100 s of μm to mm ranges), the advantage is that they are small enough to minimise the chances of unfavourable molecular contacts that would prevent them from binding efficiently. This allows fragment libraries to be constructed to sample a large chemical diversity or target‐specific interactions on the protein. To this end we screened The Scottish Hit Discovery Facility (University of Dundee) fragment library on a surface of His‐CypA created by our capture/stabilisation method. This fragment library represents a set (670 compounds) of diverse structures that comply with Astex's ‘Rule of Three’ 65, have excellent medicinal chemistry tractability and feature as key pharmacophores in a large number of bioactives.

**Figure 5:**
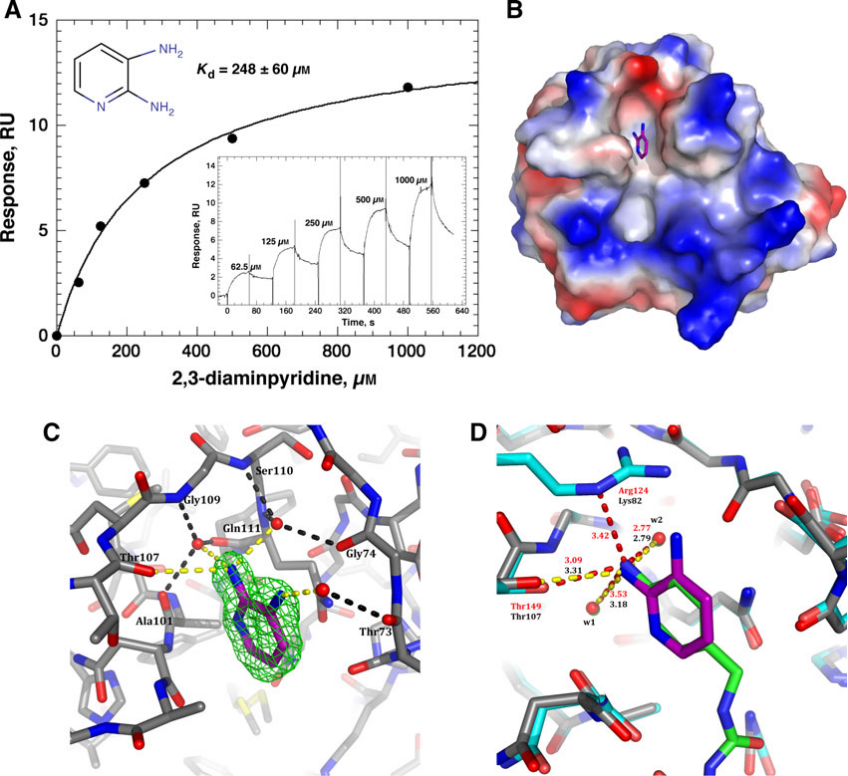
Binding and structural analysis of His‐CypA–2,3‐diaminopyridine complex. (A) Reference corrected SPR single‐cycle kinetic sensorgrams (black), monitored on a surface with 2866 RU of covalently stabilised His‐CypA (300 nm, 30‐s contact following 180‐s activation) for the indicated 2,3‐diaminopyridine concentrations (1 mm–62.5 μm). The apparent equilibrium dissociation was determined by fitting (red) a 1 : 1 Langmuir binding model (inset) to the sensorgrams using the analysis software (v2.02; GE Healthcare) supplied with the instrument. The mean *K*_d_ value is 248 ± 60 μm (*n* = 3, ±SEM). (B) Electrostatic surface of the structure of CypA in complex with 2,3‐diaminopyridine (PDB: 5LUD). The ligand is drawn with purple carbons and is observed in the Abu pocket, the hydrophobic active site is below in the orientation shown. (C) Electron density and 2,3‐diaminopyridine–CypA interaction details. The omit electron density (*F*_o_ *− F*_c_) contoured at 3σ is shown around the 2,3‐diaminopyridine ligand as a green mesh, all ligand atoms are clearly defined in density. Direct hydrogen bond interactions to the ligand are represented as yellow dashes, while bridged water–protein hydrogen bonds are represented as black dashes. (D) Comparison of the CypA–2,3‐diaminopyridine structure (grey carbons – protein, purple carbons – ligand) and the CypD–ligand structure of 4J5C (Cyan carbons – protein, Green carbons – ligand). Comparitive distances in the respective complexes are indicated (yellow dashes/black labels, CypA–2,3‐diaminopyridine; red dashes/red labels CypD–ligand structure 4J5C).

All the fragments were initially screened at 1 mm on a high‐density (to account for the mass ratio of the fragments to His‐CypA) surface of 2866 RU covalently stabilised His‐CypA‐ and cyclophilin‐A‐specific hits were further analysed with a twofold concentration series from 0.0625 to 1 mm (see Material and methods). We identified a fragment, 2,3‐diaminopyridine that bound specifically and stoichiometrically to CypA. Typical sensorgrams showing the binding of this fragment to His‐CypA are shown in Fig. 5A with an average *K*_d_ of 248 ± 60 μm. We also solved the X‐ray structure of this fragment bound to CypA to a resolution of 1.25 Å (PDB ID: 5LUD; Fig. 5B–D; Table 2). As expected, the overall structure of CypA (Fig. 5B,C) shows no major differences between the available apo‐ and ligand‐bound structures of CypA (PDB IDs 2CPL and 4N1M respectively). Electron density is present for only one copy of the ligand bound to the cyclophilin molecule (Fig. 5C) in agreement with the stoichiometric binding determined from our SPR analysis (Fig. 5A). The ligand is found bound within the small cleft, adjacent to the hydrophobic active site, commonly referred to as the Abu pocket (Fig. 5B). The electron density for the ligand is excellent, with all of its atoms well defined allowing the unambiguous positioning of the ligand in the binding cleft (Fig. 5C). The pyridine ring is sandwiched in the cleft formed by the main‐chains of alanine‐101 and asparagine‐102 on one side, and the carbonyl of glycine‐72 and the side‐chain of glutamine‐111 on the other. Only weak interactions are observed around the pyridine ring. Both amino groups point towards the back of the Abu pocket and somewhat surprisingly, only a single one – the 3‐amino group – interacts directly with the protein; the 2‐amino group forms a long range (3.31 Å) hydrogen bond to the carbonyl of threonine‐107 (Fig. 5C). The strongest interactions that the ligand makes are to three water molecules (labelled w1 to w3; Fig. 5C). These water molecules bridge interaction between the protein surface and the ligand. The 3‐amino group forms a hydrogen bond to a single water molecule (w3) which in turn bridges to the carbonyl of Threonine‐73 (Fig. 5C), while the 2‐amino group forms hydrogen bonds to two water molecules (w1 and w2; Fig. 5C). These are very well defined with each having a low B‐factor (17.64 and 17.67 Å^2^, for w1 and w2 respectively) and are reported as being well conserved in a number of cyclophilin structures 15, 68. They form an integral part of the interaction surface of the binding site of cyclophilins. Each of these water molecules further form three hydrogen bonds with the protein; w1 bonding to the main‐chain carbonyls of threonine‐107, glutamine‐111 and the main‐chain NH of glycine‐107 (Fig. 5C). w2 forms hydrogen bonds to the carbonyl of Glycine‐74 and the main‐chain NHs of serine‐110 and glutamine‐111 (Fig. 5C). The role of these well‐defined bridging water molecules reiterates that targeting water molecules rather than replacing them may in some case enhance or finesse the binding specificity and be preferable in considerations for ligand design 69, 70.

**Table 2:** X‐ray data collection and refinement statistics

X‐ray source	I02 (DLS)
Wavelength (Å)	0.97949
Space group	*P2*_*1*_ *2*_*1*_ *2*_*1*_
Unit cell (Å)	*a* = 42.53, *b* = 54.48, *c* = 86.81
Resolution range (Å)	33.95–1.25 (1.27–1.25)
Total observations	203 449
Unique reflections^a^	55 267 (2453)
Redundancy	3.7 (2.7)
*I*/σ(*I*)^a^	8.7 (1.0)
*R*_merge_ (%)^a^	6.5 (59.4)
Completeness (%)^a^	97.8 (89.5)
Refinement statistics	
Resolution limit (Å)^a^	33.0–1.25 (1.28–1.25)
*R‐*factor (%)	19.3 (31.4)
*R*_free_ (%)	21.9 (32.2)
No. of protein atoms	1303
No. of water atoms	238
No. of ligand atoms	8
RMSD bond lengths (Å)	0.0254
RMSD bond angles (°)	2.294
Mean *B* factor (Å^2^)	17.751

^a^Values in parentheses refer to the highest resolution shell.

Recently, a number of structures of submicromolar nonpeptide inhibitors of cyclophilin‐D (CypD), with evidence of inhibitory interactions with CypA, have been reported containing an aniline group bound within the Abu pocket 71. Overlaying our CypA: 2,3‐diaminopyridine structure reported here and the crystal structure of CypD in complex with one of these inhibitors (PDB ID: 4J5C) reveals a very close correlation between the aniline and the equivalent atoms of the 2,3‐diaminopyridine (the mean distance for seven equivalent atoms is 0.31, RMSD for 164 C‐alpha protein atoms is 0.437, Fig. 5D). It is noticeable that the amine group in the 4J5C structure does not have as tight an interaction to the equivalent w1 water molecule, compared to 2,3‐diaminopyridine; the distance of the equivalent bond increasing by 0.34 to 3.53 Å. The amine group in the CypD structure is near equidistant between this water and the side‐chain of arginine‐124. The equivalent side‐chain in CypA is lysine‐82, and this residue side‐chain adopts a different conformation that does not interact with the 2,3‐diaminopyridine ligand (Fig. 5D). This important difference provides a tantalising indication towards a route to isoform‐specific ligand design for CypA.

The structure of cyclophilin‐A in complex with 2,3‐diaminopyridine also validates our refined methodology for generating SPR sensor surfaces of recombinant his‐tagged human cyclophilin‐A. Furthermore, it highlights the potential role of bridging water molecules in ligand design and may provide insight for the future design of isoform‐specific cyclophilin inhibitors.

The new protocols reported here have very significantly, and rationally, optimised the basic methodology previously developed in our lab. This major protocol refinement has allowed for advancement in the biophysical characterisation of the protein and ligand binding interaction, and importantly, identification of a new nonpeptide hit/ligand for CypA. The improved activity and stability of the material on the surface of the sensors, and the ability to use much lower concentrations of a complicated ligand with solubility issues (CsA), as a direct result of this increased sensitivity and activity, allowed the measurement of the binding interaction over a much wider temperature range, than has been possible before. We were able to expand the thermo‐kinetic range by 10 °C in both directions in this new work – from 16–35 °C to 5–44 °C. This also enabled us to increase the data density across this expanded temperature range by a factor of nearly 3 (14 sets of kinetic measurements versus 5); resulting in a much more robust kinetic, affinity and thermodynamic characterisation of the binding interaction between CypA and CsA. Of note, the work presented here is the most comprehensive study of Cyp–CsA interaction by SPR to date, and was only achievable by the developments in methodology developed by our lab and described here.

## Author contribution

MAW performed the purification, designed, performed and analysed the SPR and biophysical experiments and cowrote the manuscript. MN designed the study, performed the fragment screening experiments and cowrote the manuscript. EAB performed and analysed the DLS experiments and cowrote the manuscript. IWM performed the data collection and X‐ray crystallography experiments. MDW cowrote the manuscript.
